# The increased expression and aberrant methylation of SHC1 in non–small cell lung cancer: Integrative analysis of clinical and bioinformatics databases

**DOI:** 10.1111/jcmm.16717

**Published:** 2021-06-11

**Authors:** Yicheng Liang, Yangyang Lei, Minjun Du, Mei Liang, Zixu Liu, Xingkai Li, Yushun Gao

**Affiliations:** ^1^ Department of Thoracic Surgery National Cancer Center/National Clinical Research Center for Cancer/Cancer Hospital Chinese Academy of Medical Sciences and Peking Union Medical College Beijing China; ^2^ Department of Interventional Radiology Zhongshan Hospital Fudan University Shanghai China

**Keywords:** LUAD, lung cancer, LUSC, methylation, OS, SHC1

## Abstract

Despite the previous evidence showing that SHC adaptor protein 1 (SHC1) could encode three distinct isoforms (p46SHC, p52SHC and p66SHC) that function in different activities such as regulating life span and Ras activation, the precise underlying role of SHC1 in lung cancer also remains obscure. In this study, we firstly found that SHC1 expression was up‐regulated both in lung adenocarcinoma (LUAD) and in lung squamous cell carcinoma (LUSC) tissues. Furthermore, compared to patients with lower SHC1 expression, LUAD patients with higher expression of SHC1 had poorer overall survival (OS). Moreover, higher expression of SHC1 was also associated with worse OS in patients with stages 1 and 2 but not stage 3 lung cancer. Significantly, the analysis showed that SHC1 methylation level was associated with OS in lung cancer patients. It seemed that the methylation level at specific probes within SHC1 showed negative correlations with SHC1 expression both in LUAD and in LUSC tissues. The LUAD and LUSC patients with hypermethylated SHC1 at cg12473916 and cg19356022 probes had a longer OS. Therefore, it is reasonable to conclude that SHC1 has a potential clinical significance in LUAD and LUSC patients.

## 1 INTRODUCTION

Worldwide, lung cancer is the leading cause of cancer death because it has no obvious symptoms initially and often discovered at an advanced stage.^1^ Most lung cancer patients have a poor prognosis with an overall 5‐year survival rate of 10% to 15%.^2^, ^3^ According to different histologic subtypes, lung cancer is divided into adenocarcinoma, squamous cell carcinoma, large cell carcinoma and small cell lung cancer.^4^ Of these different histologic subtypes, lung adenocarcinoma (LUAD) and lung squamous cell carcinoma (LUSC) constitute the majority of lung cancer subtypes. These two subtypes of lung cancer are derived from different cells and have some evident differences not only in biological patterns and molecular characteristics, but also in therapeutic strategies.^5^, ^6^ Ongoing research relating to the epigenetic understanding of lung cancer has made significant progress. Several comprehensive analyses of high‐throughput DNA methylation and gene expression emphasized the potential of epigenomics in clinical lung cancer classification.^7^ Aberrant DNA methylation patterns are often observed in lung carcinogenesis.^8^, ^9^ Yang et al analysed the gene expression and methylation of lung cancer and found that LUAD and LUSC showed higher differences in DNA methylation.^5^ These DNA methylation differences play key roles in medicine. Better understanding of the molecular and methylation characteristics in LUAD and LUSC will be essential in pathologic diagnosis and personalized therapy.

SHC1 (also known as SHCA) could encode three main isoforms (p46SHC, p52SHC and p66SHC) with different activities and subcellular location.^10^, ^11^ p46SHC and p52SHC are ubiquitous and stem from alternative usage of translation initiation sites within the same transcript while p66SHC, which contains a unique N‐terminal region, is considered to result from the activation of an alternative promoter.^12^ It has been shown that SHC1 products directly bind to the middle tumour (MT) antigen protein and act as an adaptor protein to activate downstream signal cascades such as RAS/MAPK and PI3K, which have a positive effect on tumorigenesis.^13^ Furthermore, the previous study showed that EGFR phosphorylation of SHC1 could stimulate Ras‐Erk MAPK and PI3K‐Akt pathways. EGFR inhibitor AG1478 could abolish phosphorylation of all Tyr and Ser/Thr sites in SHC1.^14^ It was also found that SHC1 was annotated in extracellular matrix process, which was regulated by EGFR.^15^ Over the past decades, EGFR has been identified as an oncogenic driver in non–small cell lung cancer (NSCLC). Blocking EGFR with specific tyrosine kinase inhibitors (TKIs) can produce tumour responses in NSCLC.^16^, ^17^ However, despite the evidence that EGFR plays significant roles in NSCLC, the roles of SHC1 are still unclear.

In this present study, we firstly analysed SHC1 expression by using Oncomine, Tumor Immune Estimation Resource (TIMER) and Human Protein Atlas (HPA) databases. To further validate the results from above databases, we also used quantitative real‐time polymerase chain reaction (qRT‐PCR) to validate the expression of SHC1 in NSCLC and paired adjacent normal tissues. Moreover, we comprehensively analysed the prognostic potential of SHC1 in LUAD and LUSC patients via Kaplan‐Meier (KM) Plotter and the Gene Expression Profiling Interactive Analysis 2 (GEPIA2) databases. While DNA methylation alteration is frequently observed in NSCLC and plays a critical role in carcinogenesis, diagnosis and prediction,^18^, ^19^, ^20^ we also took advantage of publicly available databases such as Wanderer and MethSurv to investigate the methylation level of SHC1 and its clinical significance in LUAD and LUSC patients. All these findings in this study revealed the significance of SHC1 in LUAD and LUSC patients. The detection of SHC1 methylation at specific probes in tumour tissues from LUAD and LUSC patients may help detect tumorigenesis at early stage. Moreover, the Kaplan‐Meier curve analysis of SHC1 methylation at specific probes and SHC1 expression revealed that SHC1 might act as a biomarker for the prognosis of these two kinds of carcinomas.

## 2 MATERIALS AND METHODS

### 2.1 Oncomine database analysis

Oncomine (www.oncomine.org) is a cancer microarray database and integrated data mining platform aimed at stimulating new discovery from genome‐wide expression analyses and comparing transcriptome data of most major cancer types with respective normal tissues.^21^, ^22^ Initially, the expression level of SHC1 was analysed by Oncomine database. The threshold was set according to the following filters: (a) Gene: SHC1; (b) P‐value: 1E‐4; (c) fold change: 2; (d) gene rank: top 10%; and (e) data type: all.

### 2.2 TIMER database analysis

TIMER (https://cistrome.shinyapps.io/timer/) is a user‐friendly web interface that enables researchers to analyse and visualize immune infiltrates across diverse cancer types. In addition to exploring the associations of tumour‐infiltrating immune cells (TIICs) with gene expression, overall survival, somatic mutation and somatic copy number alteration, this database could also analyse differential gene expression and correlation between two groups of genes. The target gene expression between adjacent normal tissue and tumour tissue could be explored in different tumour types from The Cancer Genome Atlas (TCGA) in TIMER Database. The ‘DiffExp’ module in TIMER was chosen to explore the differential expression levels of SHC1 between tumour and adjacent normal tissues. The statistical significance was determined by Wilcoxon test.^23^, ^24^

### 2.3 Evaluation of SHC1 expression level in LUAD and LUSC in the HPA database

The HPA database (http://www.proteinatlas.org) is a website with the aim to map all the human proteins in cells, tissues and organs via antibody‐based imaging, mass spectrometry‐based proteomics, transcriptomics and systems biology.^25^, ^26^ To investigate the expression and location of SHC1 in LUAD and LUSC, we analysed immunohistochemistry (IHC) data of SHC1 from the HPA database and screened out images that showed main expression trend of SHC1 in normal lung tissues and these two pathological types of lung cancer tissues using three SHC1 antibodies (HPA001844, CAB005374, CAB016305, respectively). The IHC images were directly obtained from The Human Protein Atlas website.

### 2.4 Clinical samples

To elucidate the expression levels of SHC1 in NSCLC tissues and corresponding non‐cancer tissues, the NSCLC tissue samples and paired non‐cancer tissue samples were collected from 15 NSCLC patients undergoing cancer surgical resection at our hospital between January 2019 and February 2021 (LUADs, n = 8; LUSCs = 7, respectively). All 15 cases were reassessed by two experienced pathologists independently. Adjacent non‐cancer tissues were obtained at least 5 cm away from the tumour site. After surgical excision, these tissues were quickly frozen in liquid nitrogen, then stored at −80℃ until RNA extraction. Written informed consent was signed by all patients included in the study before collecting their tissue samples. The Declaration of Helsinki was complied, and this study was approved by the Clinical Research Ethics Committee of our hospital.

### 2.5 RNA extraction and quantitative real‐time PCR

Total RNA was extracted with an RNeasy Mini Kit (Qiagen), and then, the SuperScript IV Reverse Transcriptase (Thermo Fisher Scientific: 18090010) was utilized to transcribe total RNA into cDNA. Subsequently, the quantitative real‐time PCRs were performed using the SYBR^®^ Premix Ex Taq™ II (Takara, Tli RNaseH Plus, RR820Q). Melting analysis was applied to confirm the amplification of the appropriate product. The expression of SHC1 was normalized to β‐actin. The SHC1 specific primers were 5'‐CCCGCTCAGCTCTATCCTG‐3'(forward) and 5'‐GGCAACATAGGCGACATACTC‐3' (reverse). The β‐actin primers were 5'‐GAAGAGCTACGAGCTGCCTGA‐3' (forward) and 5'‐CAGACAGCACTGTGTTGGCG‐3' (reverse). PCR amplification was done using these SHC1 specific primers, generating a 191‐bp product. The qRT‐PCR data were showed as the average of three replicates ± standard error of the mean.

### 2.6 Correlation of SHC1 expression and clinical prognosis in LUAD and LUSC with different clinicopathological factors in KM Plotter database

The KM Plotter database (www.kmplot.com) is a large database that contains gene expression data and overall survival information. Sources for the database include Gene Expression Omnibus (GEO), European Genome‐phenome Archive (EGA) and TCGA. More than 50 000 genes on survival can be assessed in 21 cancer types including breast (n = 6234), ovarian (n = 2190), lung (n = 3452) and gastric (n = 1440) cancer.^27^ Herein, the correlations between SHC1 expression and clinical prognosis in LUAD and LUSC with different clinicopathological factors were analysed by KM Plotter database. The hazard ratio (HR) with 95% confidence intervals and logrank *P*‐value were calculated.

### 2.7 Analysis of prognostic potential of SHC1 in LUAD and LUSC via KM Plotter database and GEPIA2

GEPIA2 (http://gepia2.cancer‐pku.cn/) is a comprehensive platform for analysing gene expression profiles from the Genotype‐Tissue Expression (GTEx) and TCGA projects. This platform includes 9736 tumours and 8587 normal samples which could be used to generate OS and DFS in 33 different types of cancer.^28^, ^29^ The prognostic values of SHC1 in LUAD and LUSC were assessed in GEPIA2. Comparisons of SHC1 in normal tissues and lung cancer tissues were analysed and visualized in a box plot, in which logrank *P*‐value and HR with 95% were calculated and showed on the webpage. Furthermore, the correlations between SHC1 and survival in LUAD and LUSC patients were also analysed in KM Plotter database using the affymetrix ID ‘201469_s_at’. *P* < 0.05 was considered statistically significant.

### 2.8 Comprehensive analysis of SHC1 methylation in LUAD and LUSC

As epigenetic changes through DNA methylation are critical in tumorigenesis, the methylation levels of SHC1 in lung tissues and normal tissues were firstly investigated in Wanderer database. Wanderer (http://maplab.imppc.org/wanderer/) is an intuitive web tool which can be used to access and visualize gene expression and DNA methylation profiles from TCGA. The user is allowed to browse any HumanMethylation450 probes that located in the displayed genome region to explore its correlation with the gene expression values.^30^ Through the analysis of the TCGA LUAD and LUSC data sets, the changes in SHC1 methylation were obtained. The correlations between SHC1 gene expression and methylation changes were tested using the Spearman method. The strength of correlation was calculated as (*r*), and the methylation level was presented as beta value. Furthermore, to perform survival analysis based on single CpG methylation in lung cancer patients, the MethSurv tool was also utilized. MethSurv (https://biit.cs.ut.ee/methsurv/) is a valuable platform to perform survival analysis using gene expression data from TCGA for a CpG located in or around the proximity of a query gene. The univariable and multivariable survival analysis could be performed based on patient methylation levels for any CpG site (probe) using Cox proportional hazards models in MethSurv. The level of probe methylation was used as the explanatory variable and the survival time as the response variable in univariable survival analysis. In order to evaluate the differences in survival, the methylation levels of patients were divided into higher (the methylation *β* value higher than the cut‐off point; shown in red) and lower groups (the methylation *β* value lower than the cut‐off point; shown in blue).^31^ *P* < 0.05 was considered statistically significant.

### 2.9 Statistical analysis

The expression levels of SHC1 between lung cancer tissues and normal tissues were firstly evaluated by the Oncomine database and TIMER database. The results generated in Oncomine database were showed with *P*‐value, fold change, gene rank and data type. The cell colour was determined by the best gene rank percentile for the analyses within the cell in the results of Oncomine database. The results of TIMER were displayed using box plots, with statistical significance of differential expression evaluated using the Wilcoxon test. The comparative delta‐Ct method algorithms were used in qRT‐PCR. The correlation of SHC1 mRNA expression and clinical prognosis in LUAD and LUSC with different clinicopathological factors by Kaplan‐Meier Plotter database were displayed with HR, logrank *P*. The survival analyses were estimated using the Kaplan‐Meier method with a logrank test in GEPIA2 and Kaplan‐Meier Plotter database. The results in Wanderer database provided detailed individual beta values of all the HumanMethylation450 probes inside or in the vicinity of the gene. The statistical comparisons were performed to identify differential DNA methylation between normal and tumour samples at the single probe level. Wanderer also performed Wilcoxon rank sum test on normal versus tumour provided there were at least two observations in each group. The results in MethSurv web portal were displayed containing information related to HR and log‐likelihood ratio (LR) test *P*‐value. *P* < 0.05 were considered statistically significant.

## 3 RESULTS

### 3.1 The expression level of SHC1 was elevated in lung cancer tissues in bioinformatics database

**FIGURE 1:**
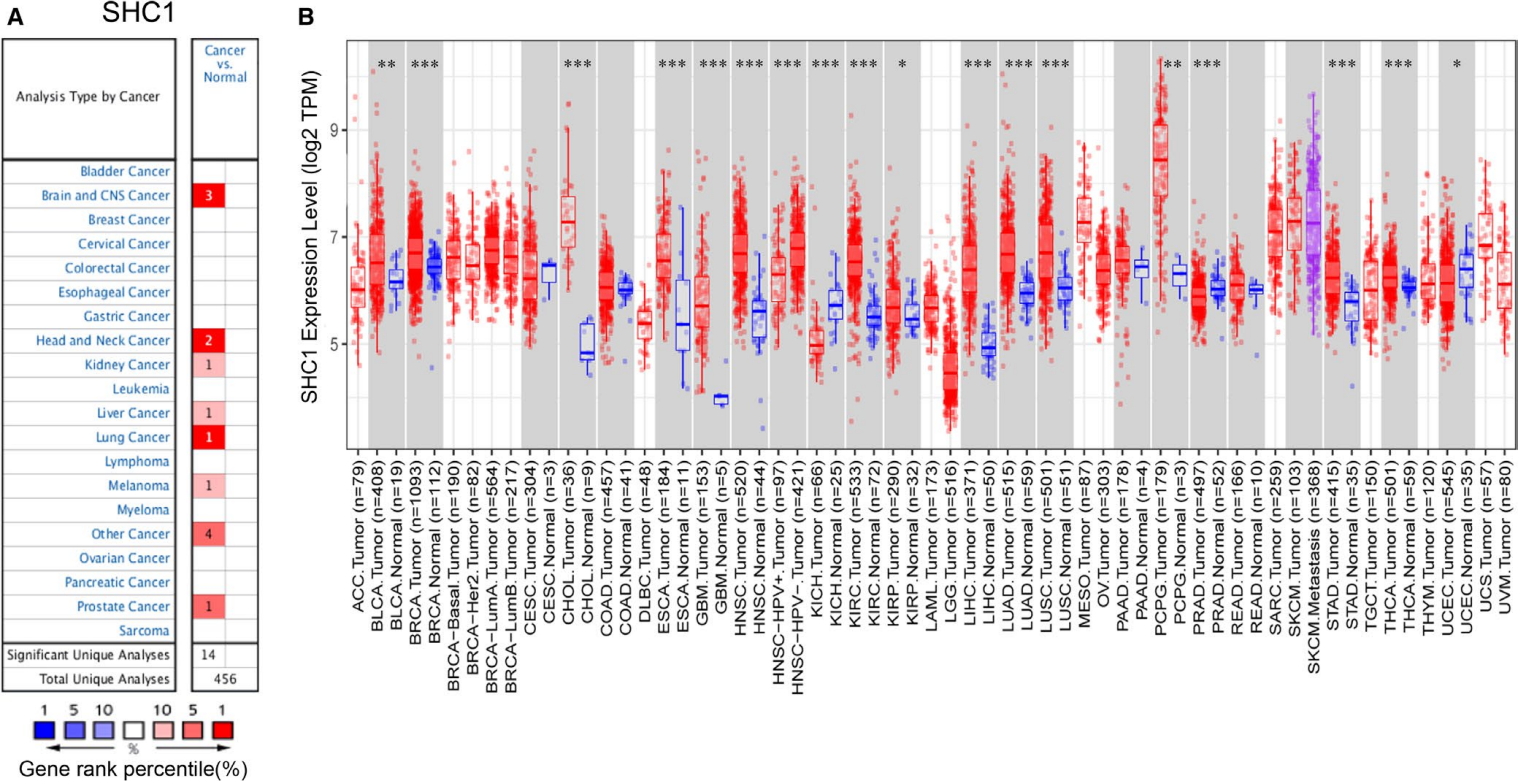
SHC1 expression levels in different cancer types. A, The graph demonstrated the overexpression (red) or down‐regulation (blue) of SHC1 in different data sets (Cancer vs. Normal tissue). Cell colours indicated the best gene rank percentile for analyses. B, SHC1 expression levels in different types of human cancer and normal tissues from TCGA analysed by TIMER (*P < 0.05, **P < 0.01, ***P < 0.001). TCGA, The Cancer Genome Atlas; TIMER, Tumor Immune Estimation Resource

**FIGURE 2:**
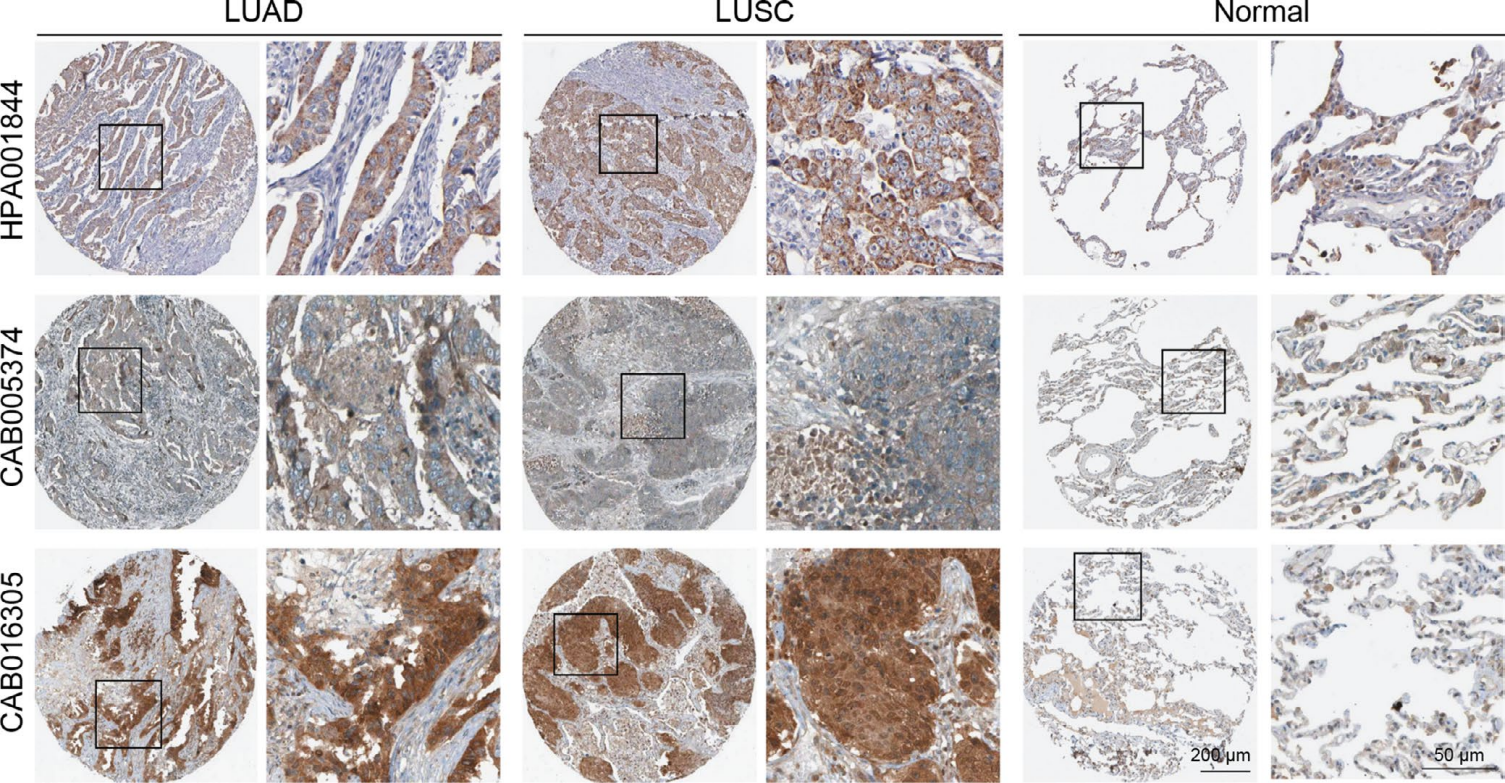
Validation the protein expression levels of SHC1 in LUAD and LUSC compared with normal lung tissues in The Human Protein Atlas database. SHC1 protein showed weak cytoplasmic/membranous staining in normal alveolar cells (antibody: HPA001844, CAB005374 and CAB016305, respectively) while medium or even high expression levels of SHC1 were observed in most cytoplasmic/membranous cells in LUAD and LUSC patients (antibody: HPA001844, CAB005374 and CAB016305, respectively). Representative IHC images of staining intensities of SHC1 in LUAD and LUSC compared with normal lung tissues (A‐D). IHC, immunohistochemistry; LUAD, lung adenocarcinoma; LUSC, lung squamous cell carcinoma

To investigate the different expression levels of SHC1 in normal and lung cancer tissues, the Oncomine database and TIMER database were applied. These results revealed that the mRNA expression levels of SHC1 were higher in lung cancer tissues compared to normal tissues in Oncomine database (Figure 1A). Moreover, the RNA‐seq data from TCGA in TIMER also showed that SHC1 mRNA expression levels were all obviously elevated in LUAD and LUSC tissues when compared to normal tissues (Figure 1B). The IHC data from the HPA database also indicated that the protein expression level of SHC1 showed low staining in the alveolar cells of normal lung tissues, whereas medium or even high expression levels of SHC1 were observed in most LUAD and LUSC tissues by using the antibodies HPA001844, CAB005374 and CAB016305, respectively (Figure 2). In summary. These results revealed that the expression level of SHC1 was overexpressed in LUAD and LUSC tissues.

### 3.2 The expression of SHC1 mRNA was significantly higher in NSCLC tissues than the paired non‐cancer tissues in clinical samples

**FIGURE 3:**
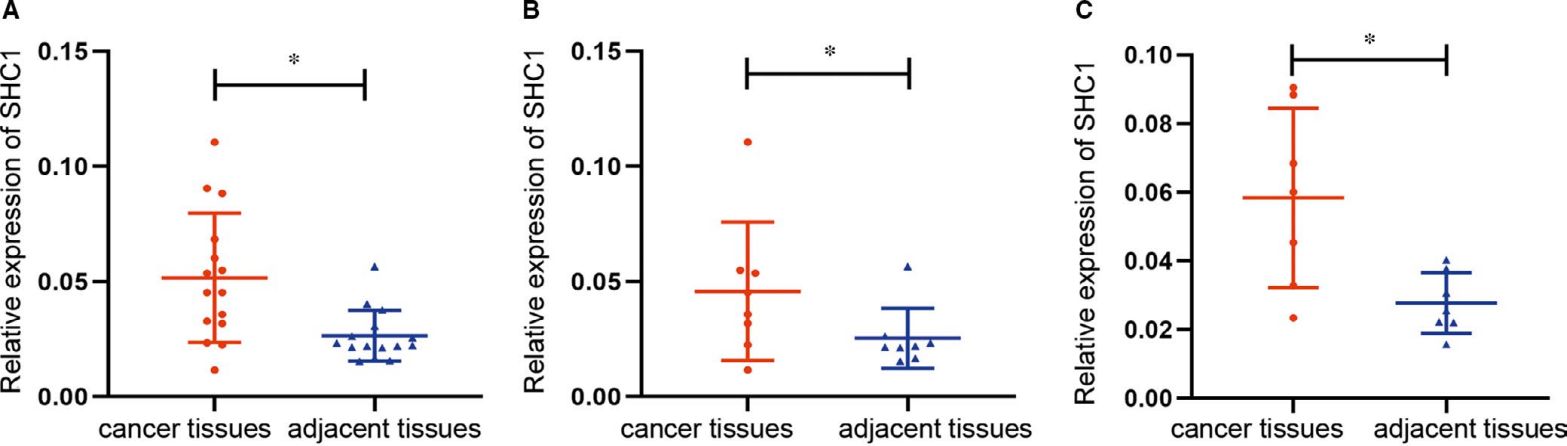
qRT‐PCR analysis of SHC1 mRNA expression in 15‐paired tumour and non‐tumour lung tissues (A‐C). A, SHC1 expression was up‐regulated in LUAD and LUSC (LUADs, n = 8; LUSCs=7, respectively). B, SHC1 expression was up‐regulated in LUAD (n = 8). C, SHC1 expression was up‐regulated in LUSC (n = 7). qRT‐PCR, quantitative real‐time polymerase chain reaction; LUAD, lung adenocarcinoma; LUSC, lung squamous cell carcinoma

The qRT‐PCR analyses indicated that the expression level of SHC1 mRNA was up‐regulated in the majority of NSCLC tissues than that in adjacent noncancerous tissues (*P* < 0.05) (Figure 3A). The qRT‐PCR analyses also showed that SHC1 mRNA level increased significantly in LAUD tissues compared to adjacent tissues (*P* < 0.05) (Figure 3B). Moreover, the expression level of SHC1 mRNA was also up‐regulated in LUSC tissues compared to adjacent tissues (*P* < 0.05) (Figure 3C).

### 3.3 The correlation of SHC1 expression and clinical prognosis in lung cancer with different clinicopathological factors

In order to explore the relevance and underlying mechanisms of SHC1 expression in lung cancer, we investigated the different prognostic factors in lung cancer via the KM Plotter database. Overexpression of SHC1 was correlated with poorer OS in female lung cancer patients (OS HR = 1.79, logrank *P* < 0.01). Furthermore, high expression of SHC1 was also associated with worse OS in stages 1 and 2 but not stage 3 lung cancer patients (OS HR = 1.86, logrank *P* < 0.01; OS HR = 1.7, logrank *P* = 0.0041; OS HR = 0.94, logrank *P* = 0.8145). A total of 577 lung cancer patients with higher SHC1 expression were stage 1 at diagnosis (577/891, 64.76%). In addition, high SHC1 expression lung cancer patients had poorer OS in the American Joint Committee on Cancer (AJCC) stage M0 (OS HR = 1.31, logrank *P* = 0.0114) while the patient number was not enough for meaningful analysis in AJCC stage M1 (Table 1). These data indicated that SHC1 expression can be detected even in patients with stage 1 cancer. SHC1 expression level may influence the OS in LUAD patients and the gender, stage, and metastasis stage were also the significant prognostic factors in lung cancer patients.

**TABLE 1:** Analysis of the correlation between SHC1 mRNA expression and clinical prognosis in lung cancer patients with different clinicopathological factors by using Kaplan‐Meier plotter

Clinicopathological characteristics	Overall survival n = 1927		
	N	Hazard ratio	*P*‐value
Histology			
Adenocarcinoma	719	1.9 (1.49‐2.42)	1.10E‐07
Squamous cell carcinoma	524	1.06 (0.84‐1.35)	0.62
Stage			
1	577	1.86 (1.41‐2.46)	7.90E‐06
2	244	1.7 (1.18‐2.46)	0.0041
3	70	0.94 (0.54‐1.63)	0.8145
Grade			
I	201	1.01 (0.71‐1.45)	0.947
II	310	1.04 (0.76‐1.43)	0.7922
III	77	1.43 (0.74‐2.76)	0.2898
AJCC stage T			
1	437	1.21 (0.91‐1.6)	0.1939
2	589	0.99 (0.79‐1.23)	0.91
3	81	1.05 (0.64‐1.72)	0.8601
4	46	1.02 (0.54‐1.93)	0.9491
AJCC stage N			
0	781	0.99 (0.8‐1.22)	0.9319
1	252	1.15 (0.84‐1.58)	0.3701
2	111	0.98 (0.65‐1.47)	0.9123
AJCC stage M			
0	681	1.31 (1.06‐1.62)	0.0114
1	No		
Gender			
Female	714	1.79 (1.41‐2.27)	1.10E‐06
Male	1100	1.13 (0.96‐1.32)	0.1339
Chemotherapy			
No	310	0.93 (0.67‐1.31)	0.6912
Yes	176	1.31 (0.86‐2.01)	0.2085
Radiotherapy			
No	271	1.04 (0.73‐1.48)	0.8363
Yes	70	1.08 (0.63‐1.83)	0.7804

Note

*P* < 0.05 was statistically significant.

### 3.4 The prognostic value of SHC1 in LUAD and LUSC patients

**FIGURE 4:**
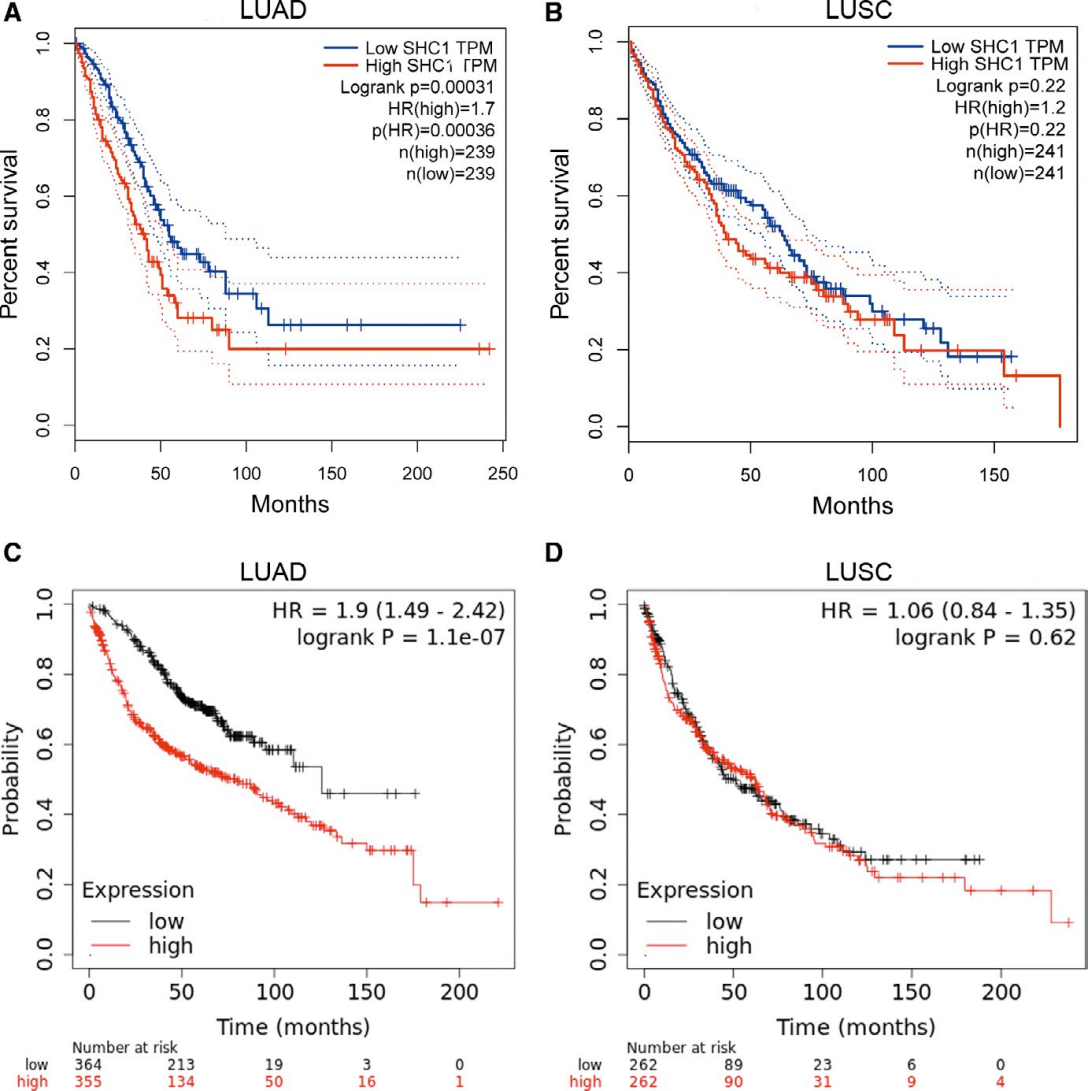
Kaplan‐Meier curves for overall survival based on SHC1 expression levels of LUAD and LUSC patients in GEPIA2 and Kaplan‐Meier Plotter databases (A‐D). A‐B, Survival curves of LUAD and LUSC patients in GEPIA2 database (n = 478, n = 482, respectively). Overexpression of SHC1 predicted poorer OS in LUAD patients in GEPIA2 database (HR = 1.7, logrank *P* = 0.00031). C‐D, Survival curves of LUAD and LUSC patients in Kaplan‐Meier Plotter database (n = 719, n = 524, respectively). Overexpression of SHC1 predicted poorer OS in LUAD patients in KM Plotter database by using ‘201469_s_at’ affymetrix ID (HR = 1.9, logrank *P* = 0.01). GEPIA2, Gene Expression Profiling Interactive Analysis 2; OS, overall survival

The relationship between SHC1 expression and survival in LUAD and LUSC patients was investigated in GEPIA2 and KM Plotter database. The results in GEPIA2 and KM Plotter database showed that high expression of SHC1 was only associated with poorer OS in LUAD patients (OS HR = 1.7, logrank *P* = 0.00031; OS HR = 1.9, logrank *P* < 0.01) but not in LUSC patients (OS HR = 1.2, logrank *P* = 0.22; OS HR = 1.06, logrank *P* = 0.62) (Figure 4). These results suggested that SHC1 expression has an impact on the prognosis of LUAD patients.

### 3.5 DNA methylation of SHC1 in LUAD and LUSC data sets

**FIGURE 5:**
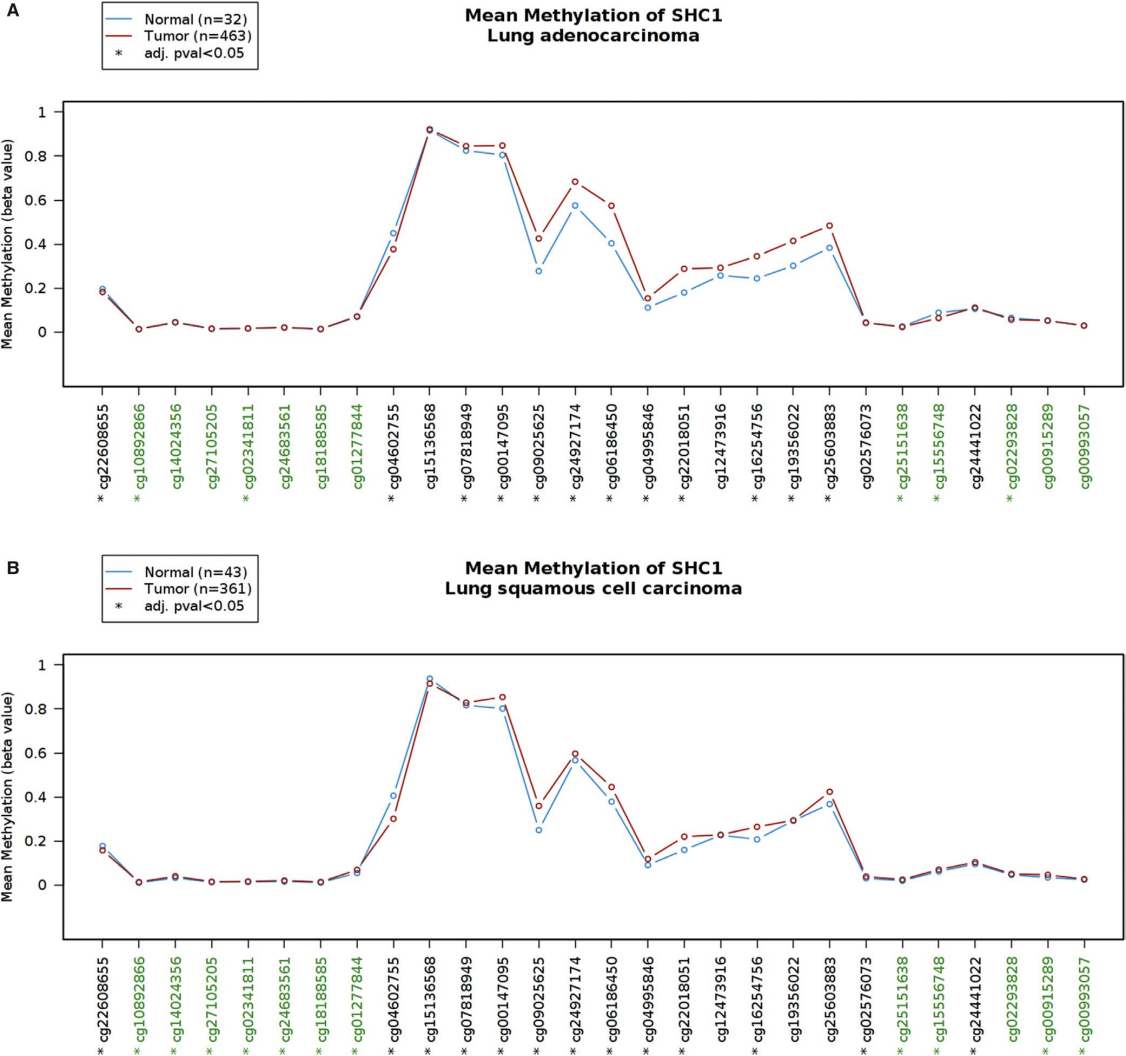
Analysis of SHC1 methylation level at different probes in LUAD and LUSC datasets by using 450k methylation array in Wanderer database. The graph allowed the comparison of tumour and normal profiles. A, In LUAD data set, probe‐level analysis revealed 10 evident hypermethylated probes in SHC1 flanking regions in LUAD tissues (cg07818949, cg00147095, cg09025625, cg24927174, cg06186450, cg04995846, cg22018051, cg16254756, cg19356022, cg25603883, respectively); in SHC1 CpG island (green probes), 5 probes (cg10892866, cg02341811, cg25151638, cg15556748 and cg02293828) also showed differences in methylation level between LUAD tissues and normal tissues (*P < 0.05). B, In LUSC data set, probe‐level analysis identified nine evident hypermethylated sites in SHC1 flanking regions in LUSC tissues (cg07818949, cg00147095, cg09025625, cg24927174, cg06186450, cg04995846, cg22018051, cg16254756, cg02576073, respectively); in SHC1 CpG island (green probes), cg10892866, cg14024356, cg27105205, cg02341811, cg24683561, cg18188585, cg01277844, cg25251638, cg15556748, cg00915289 and cg00993057 showed differences in methylation level between LUSC tissues and normal tissues (*P < 0.05). The plot and *P* values were produced using Wanderer (http://maplab.imppc.org/wanderer/)

To explore the underlying mechanism leading to abnormal expression of SHC1, the level of whole SHC1 methylation in 463 LUAD tissues and 32 normal tissues as well as 361 LUSC tissues and 43 normal tissues from TCGA data for methylation arrays in Wanderer database were assessed. There were totally 28 probes in the selected region of SHC1 (154 933 000‐154 947 000) in LUAD and LUSC data sets. In LUAD data set, probe‐level analysis identified 10 evident hypermethylated probes in the selected region of SHC1 in LUAD tissues (cg07818949, cg00147095, cg09025625, cg24927174, cg06186450, cg04995846, cg22018051, cg16254756, cg19356022 and cg25603883, *P* < 0.05); in CpG islands (CpG islands were displayed in green colour in the *x*‐axis), cg10892866, cg02341811, cg25151638, cg15556748 and cg02293828 also showed differences in methylation level between LUAD tissues and normal tissues (*P* < 0.05). Among these above five CpG islands in LUAD, we found that cg15556748 showed evidently hypermethylated in normal tissues than LUAD tissues (Figure 5A). Furthermore, in LUSC data set, probe‐level analysis identified nine evident hypermethylated probes in the selected region of SHC1 in LUSC tissues (cg07818949, cg00147095, cg09025625, cg24927174, cg06186450, cg04995846, cg22018051, cg16254756 and cg02576073, *P* < 0.05); in CpG islands, cg10892866, cg14024356, cg27105205, cg02341811, cg24683561, cg18188585, cg01277844, cg25251638, cg15556748, cg00915289 and cg00993057 showed differences in methylation level between LUSC tissues and normal tissues(*P* < 0.05) (Figure 5B).

**FIGURE 6:**
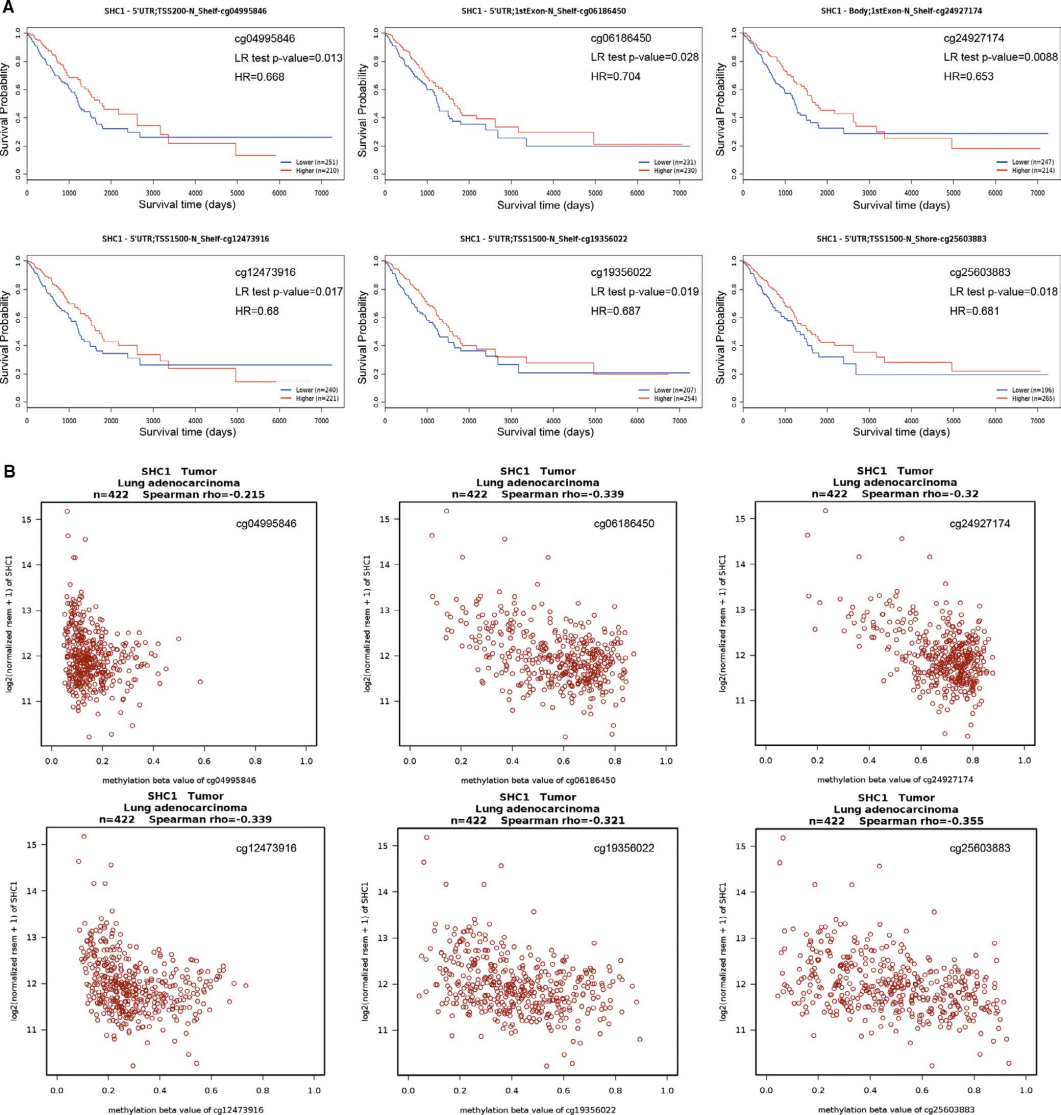
Analysis of the relationships among SHC1 methylation levels at specific probes, survival time and SHC1 expression level in LUAD data set. A, Kaplan‐Meier plots containing the correlations between survival time and the methylation status of SHC1 at specific probes in LUAD data set. B, The correlation between SHC1 expression and the methylation status of SHC1 at specific probes in LUAD data set. The Kaplan‐Meier plots and graphs were produced using MethSurv tool (https://biit.cs.ut.ee/methsurv/)

**FIGURE 7:**
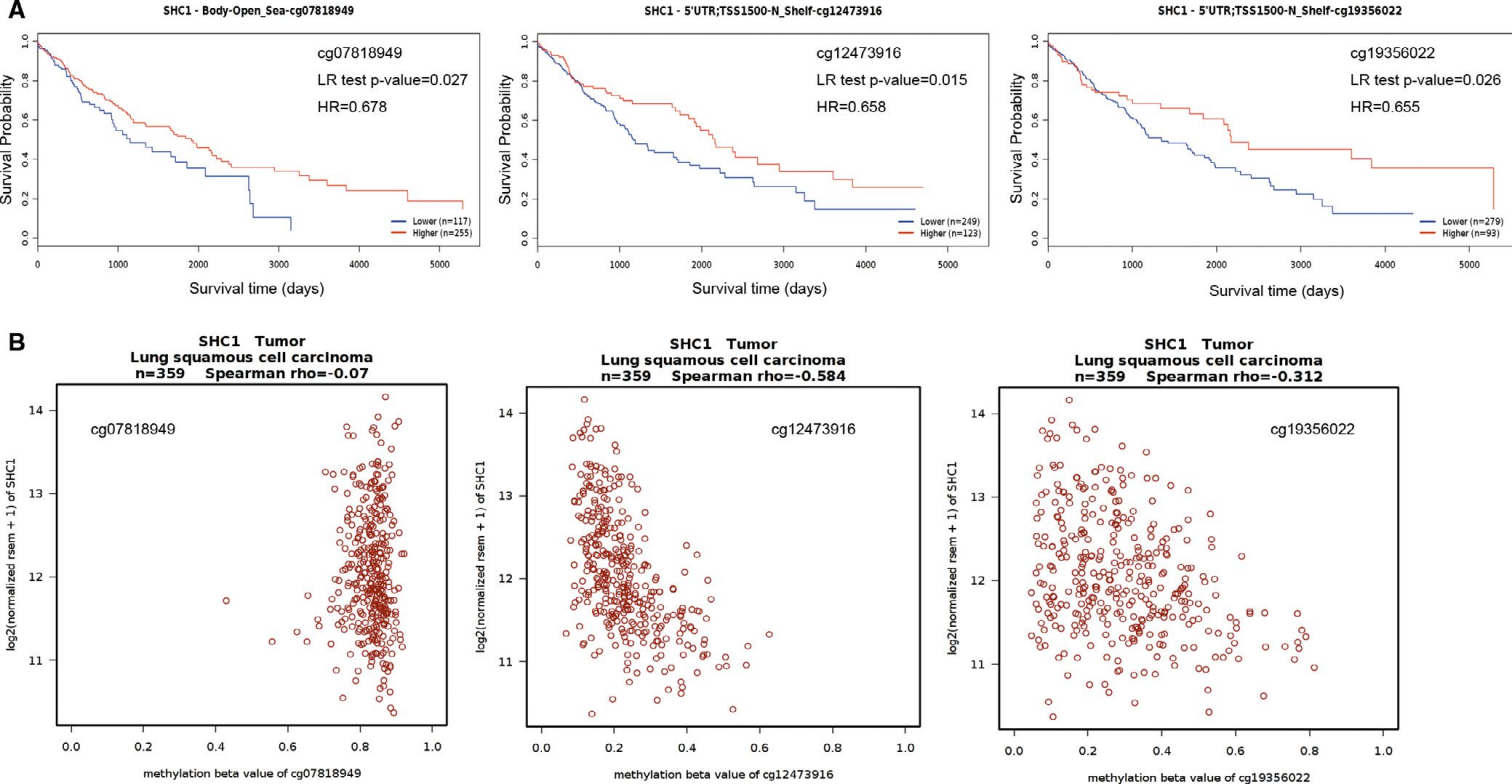
Analysis of the relationships among SHC1 methylation levels at specific probes, survival time and SHC1 expression level in LUSC data set. A, Kaplan‐Meier plots containing the correlations between survival time and the methylation status of SHC1 at specific probes in LUSC data set. B, The correlation between SHC1 expression and the methylation status of SHC1 at specific probes in LUSC data set

In order to further investigate the association between the methylation level of each probe and survival rate in LUAD and LUSC patients, the MethSurv database was used with probe methylation levels as explanatory variable and survival time as the response variable. The Kaplan‐Meier plots in MethSurv database showed that the methylation level of cg04995846, cg06186450, cg24927174, cg12473916, cg19356022 and cg25603883 was associated with survival time in LUAD patients; LUAD patients with higher methylation level of these probes had a longer OS (*P* = 0.013, HR = 0.668; *P* = 0.028, HR = 0.704; *P* = 0.0088, HR = 0.653; *P* = 0.017, HR = 0.68; *P* = 0.019, HR = 0.687; *P* = 0.018, HR = 0.681, respectively) (Figure 6A). In addition, the correlations between methylation levels of these probes and SHC1 expression in Wanderer database showed that SHC1 expression was negatively associated with methylation level of these probes in LUAD tissues (Spearman rho = −0.215; −0.339; −0.32; −0.339; −0.321; −0.355, respectively) (Figure 6B). Furthermore, the Kaplan‐Meier plots in MethSurv database showed that the methylation level of cg07818949, cg12473916 and cg19356022 was associated with survival time in LUSC patients; LUSC patients with higher methylation level of these probes had a longer OS (*P* = 0.027, HR = 0.678; *P* = 0.015, HR = 0.658; *P* = 0.026, HR = 0.655, respectively) (Figure 7A). Among these above three probes, we found that SHC1 expression was negatively associated with the methylation level of cg12473916 and cg19356022 in LUSC tissues (Spearman rho = −0.584; −0.312) (Figure 7B). It was observed that the methylation level of cg12473916 and cg19356022 showed negative correlations with SHC1 expression both in LUAD and in LUSC tissues. In addition, the LUAD and LUSC patients with hypermethylated cg12473916 and cg19356022 had a longer OS, indicating that hypermethylation of SHC1 at cg12473916 and cg19356022 probes could influence the prognosis both in LUAD and LUSC patients.

## 4 DISCUSSION

Nowadays, lung cancer also remains the leading cause of cancer‐related death wordwide.^32^ Despite significant advances have been achieved, further improvements are also needed in lung cancer patients to enable diagnosis at the early stage of the disease. Screening for the potential biomarkers of lung cancer makes the diagnosis possible at the early stage.^33^ NSCLC is a heterogeneous class of tumours, accounting for nearly 85% of all newly diagnosed lung cancer patients.^34^ Epigenetic alterations, such as DNA methylation, have been thought to be closely related to tumorigenesis and progression.^35^ Several DNA methylation changes were associated with lung cancer risk.^36^, ^37^ Recently, it was found that p66SHC, one of the SHC1 gene encoding proteins, whose epigenetic enhancement was related to the increased histone acetylation and methylation specifically.^38^ Although accumulating evidence clarifies that the abnormal overexpression of SHC1 may indicate the worst expected outcome in diverse types of cancers, including breast cancer^10^ and clear cell renal cell carcinoma,^15^ they neglect the impact of SHC1 expression level on NSCLC patients. We observed that the expression level of SHC1 showed higher expressed both in LUAD and in LUSC tissues in publicly available databases. Moreover, the qRT‐PCR analysis also showed relatively high SHC1 expression in LUAD and LUSC tissues compared to adjacent tissues. Besides, higher SHC1 was associated with a poorer OS in LUAD patients. The gender, stage and metastasis stage were also the significant prognostic factors in lung cancer patients by analysing the data in Kaplan‐Meier Plotter database. Overexpression of SHC1 was correlated with poorer OS in female lung cancer patients as well as the patients with stages 1 and 2 but not stage 3 lung cancer. In addition, high SHC1 expression lung cancer patients had poorer OS in AJCC stage M0. Therefore, high‐expressed SHC1 may influence OS in LUAD patients and the gender, stage and metastasis stage were also the significant prognostic factors in lung cancer patients. Additionally, our data revealed that SHC1 methylation was associated with OS in lung cancer patients although multiple probes showed individual differences. The aberrant methylation levels were calculated independently for analysis. The results showed that the methylation level of cg04995846, cg06186450, cg24927174, cg12473916, cg19356022 and cg25603883 within SHC1 was associated with survival time in LUAD patients. Furthermore, the methylation level of cg07818949, cg12473916 and cg19356022 was associated with survival time in LUSC patients. The LUAD and LUSC patients with hypermethylated cg12473916 and cg19356022 within SHC1 had a longer OS. Meanwhile, it was observed that the methylation levels of cg12473916 and cg19356022 showed negative correlations with SHC1 expression both in LUAD and in LUSC tissues. These findings suggested that the expression level of SHC1 and aberrant methylation levels at specific probes within SHC1 could be effective biomarkers for lung cancer patients. As previous studies indicated that both gene expression and methylation profiles explained significant differences in survival,^39^ the combination of SHC1 expression with the methylation levels at cg12473916 and cg19356022 would provide better prognostic information in LUAD and LUSC patients.

The heterogeneity of lung cancer is of great significance in the understanding of pathogenesis, diagnosis and therapeutic decision. Despite recent developments in lung cancer diagnosis and therapy, the most challenge was also closely related to the complex molecular and histological characterizations of lung cancer.^40^ Epigenetic modifications such as DNA methylation and histone modification, regulate gene expression, which in turn play a decisive role in dynamic molecular and cellular events in lung cancer evolution.^41^ Gene expression and DNA methylation data have begun to reveal heterogeneity in lung cancer tissues.^42^, ^43^ DNA methylation is an important epigenetic mechanism that plays an important role in human health.^44^ In contrast to normal cells of the same tissue type, two types of DNA methylation changes appear to occur in tumour cells including demethylation within many regions of the genome and de novo methylation of select CpG islands.^45^ It is widely known that some tumour‐suppressor gene inactivation is caused by hypermethylation within the promoter region, and many previous studies revealed that in different cancer types, many associated genes are silenced by DNA methylation.^46^ Aberrant DNA methylation can occur through mutations before or following cell transformation.^45^ Owing to improved genome‐scale mapping of methylation, DNA methylation can be evaluated in different genomic contexts, including transcriptional start sites with or without CpG islands, in gene bodies, at regulatory elements and at repeat sequences. It seems that the function of DNA methylation varies with context.^47^ As DNA methylation changes often occur at the early stages of cancer initiation, the assessment of the DNA methylation may provide a very promising area of new cancer biomarker discovery in early diagnosis. Accumulating evidence indicated that DNA methylation was the secondary ‘motive’ for promoting oncogenesis following the genetic mutations, which also revealed that DNA methylation may be an important biomarker for early detection of tumours.^48^, ^49^, ^50^

DNA hypermethylation emerges as a hallmark in lung cancer and often occurs at early stage in carcinogenesis.^51^ Distinct methylation patterns can contribute to molecular distinctions between different histologic subtypes of lung cancer.^52^ It was also demonstrated that the development of pro‐metastatic phenotypes of NSCLC is related to changes in genome‐wide DNA methylation.^53^ In conclusion, epigenetic events play the pivotal role in lung cancer pathogenesis. LUAD and LUSC are the main histological types of NSCLC. The mechanisms underlying LUAD and LUSC indicated that these two histological types were caused by diverse dysfunctional pathways.^54^ Further understanding of the differences between the gene expression and methylation patterns in these two histological types of lung cancer may promote the development of new strategies for the early prevention, diagnosis and treatment of lung cancer patients.^5^ SHC1 is an ubiquitously expressed adaptor protein which exists in three isoforms (p46SHC, p52SHC and p66SHC).^11^ Previous studies revealed that EGFR activation in NSCLC cells could release SHC‐binging protein 1 (SHCBP1) from SHC1, which subsequently transferred to the nucleus and advanced the trans‐activation of β‐catenin, thus leading to the development of stemness and malignant progression in NSCLC cells.^55^ Furthermore, it was reported that SHC1 was associated with cancer development and progression through regulating cell proliferation, apoptosis and migration in bladder cancer.^56^ Moreover, many studies revealed that SHC1 participated in signalling through epidermal growth factor receptor‐2 (HER‐2), oestrogen receptor (ER) and prolactin (PR) signalling, which were well‐recognized biological markers for predicting prognosis and response to breast cancer therapy.^57^, ^58^, ^59^ However, very little is known about the role of SHC1 in lung cancer. In conclusion, we highlighted SHC1 clinical significance and its methylation in lung cancer. SHC1 was found to be higher expressed both in LUAD and in LUSC patients. Furthermore, SHC1 were aberrantly methylated at specific probes in LUAD and LUSC. Our results revealed that these two subtypes of lung cancer showed higher differences in SHC1 methylation. As changes in DNA methylation often occur at early stage of cancer development, DNA methylation–based biomarkers show a bright future for cancer detection.^60^ Specific SHC1 methylation patterns may indicate different NSCLC subtypes. Furthermore, survival analysis for SHC1 expression and methylation indicated that SHC1 could serve as a prognostic biomarker in lung cancer patients. Although our data validated that the altered expression and methylation of SHC1 could be applied as significant markers in LUAD and LUSC patients, there was still room for improvement in our study. Given the NSCLC is a heterogeneous disorder which consists of distinct molecular subtypes, more sophisticated methods will be needed to explore the application value of SHC1 expression and methylation in early diagnosis and prognosis of LUAD and LUSC patients. In addition to demonstrating the clinical significance of methylation and higher SHC1 expression in LUAD and LUSC patients, the direct correlation with SHC1 expression and the role of methylation in distinct SHC1 region in LUAD and LUSC also deserve further investigation.

## CONFLICT OF INTEREST

All the authors in this study confirm that there are no conflicts of interest.

## AUTHOR CONTRIBUTION

**Yicheng Liang:** Conceptualization (lead); Formal analysis (lead); Methodology (lead). **Yangyang Lei:** Methodology (equal); Project administration (equal); Supervision (equal); Validation (equal); Writing‐original draft (lead). **Minjun Du:** Data curation (equal); Resources (equal); Writing‐original draft (supporting). **Mei Liang:** Validation (equal); Writing‐original draft (supporting). **Zixu Liu:** Project administration (supporting); Writing‐original draft (supporting). **Xingkai Li:** Supervision (supporting). **Yushun Gao:** Funding acquisition (lead); Project administration (lead); Writing‐review & editing (lead).

## Data Availability

The publicly available data sets were analysed in this study. The authors confirm that these data can be found here: www.oncomine.org; https://cistrome.shinyapps.io/timer/; http://www.proteinatlas.org; www.kmplot.com; http://gepia2.cancer‐pku.cn/; http://maplab.imppc.org/wanderer/; https://biit.cs.ut.ee/methsurv/. The IHC data from the HPA database can be directly obtained from https://www.proteinatlas.org/ENSG00000160691‐SHC1/tissue/lung and https://ww.proteinatlas.org/ENSG00000160691‐SHC1/pathology/lung+cancer#imid_4114164 (antibody HPA001844, CAB005374, CAB016305).
